# Removal of Common Bile Ducts Stones via Percutaneous Access with a Flexible Ureteroscope and Laser Assistance

**DOI:** 10.1155/2019/4684631

**Published:** 2019-10-15

**Authors:** R. J. L. F. Loffeld, J. Paulus, S. Langbein, B. Jansen

**Affiliations:** ^1^Department of Gastroenterology, Zaans Medisch Centrum, Zaandam, Netherlands; ^2^Department of Urology, Zaans Medisch Centrum, Zaandam, Netherlands; ^3^Department of Interventional Radiology, Zaans Medisch Centrum, Zaandam, Netherlands

## Abstract

Two patients are described with large stones in the common bile duct. Standard ERCP was not possible. Both patients were successfully treated with percutaneous access and use of the ureteroscope with the holmium laser.

## 1. Introduction

Gallbladder stones are a common diagnosis in general practice. Patients may also develop stones in the common bile duct. In addition, these can occur many years after previous cholecystectomy. The treatment of choice, especially since the introduction of the laparoscopic cholecystectomy is endoscopic retrograde cholangiography (ERC) with papillotomy and removal of stones. This procedure is successful in a high percentage of cases.

Sometimes local anatomy has changed and it is impossible to perform an ERC. A percutaneous procedure with canulation of the bile ducts, dilatation of the papilla, and pushing the stones towards the duodenum can be done.

In the present report, we describe two patients with large stones in the common bile duct and changed local anatomy, in whom stones were successfully removed using the urologic ureteroscope.

### 1.1. Case 1

The first patient is a woman of 77 years. At the age of 17 years, she underwent a Billroth II resection because of a bleeding peptic ulcer. Her medical history revealed breast cancer and cholecystectomy. One year before the present presentation, she was admitted because of colics and nausea. Blood analysis showed cholestatic liver tests: ASAT 507 U/l (reference < 30 U/l); ALAT 479 U/l (reference < 35 U/l); Total bilirubin 24 *µ*mol/l (reference < 21 *µ*mol/l); GGT 328 U/l (reference < 40 U/l); Alkaline Phosphatase 128 U/l (reference < 120 U/l), in addition there was a leucocytosis of 24.3 × 10*E*9/l (reference 4–10 × 10*E*9/l) and a CRP of 60 mg/l (reference < 5 mg/l).

CT‐scan showed dilated intra‐ and extra‐hepatic bile ducts. The diameter of the common bile duct was 1.6 cm. ERC failed because of the changed anatomy. It was impossible to introduce the endoscope in the proximal part of the duodenum. Blood culture was positive for *E*. *coli*, and she was treated accordingly with antibiotics. Percutaneous cholangiography (PTC) showed no stones (anymore!). Nevertheless balloon dilatation of the papilla was done.

One year later, she was admitted because of recurrent cholangitis. This time the bilirubin level was 131 *µ*mol/l. CT‐scan again showed dilated intra‐ and extra‐hepatic bile ducts. Blood cultures were positive for *E. coli* and *K. pneumoniae*. Because of good clinical response and decrease of the bile duct dilatation, a conservative approach with antibiotics was chosen. However, after less than one month cholangitis recurred and a PTC was done. Several small stones in the common bile duct were present. An internal biliary drain was placed for drainage. Balloon dilatation of the papilla was successful, but the stones could not be pushed through the papilla. Because of the impossibility to perform ERC (also a rendez-vous procedure failed) and the relative failure of the PTC, a percutaneous approach with a flexible ureteroscope was performed. During this procedure several stones were visualized and fragmented with the help of the holmium laser. The stone fragments were successfully removed through the dilated papilla via flushing with saline 0.9% (in total 3 L). A cholangiography several days later showed a small residual stone, which was pushed into the duodenum with a balloon. The patient recovered uneventfully and was doing well without complaints and normal blood work two months later.

### 1.2. Case 2

**Figure 1:**
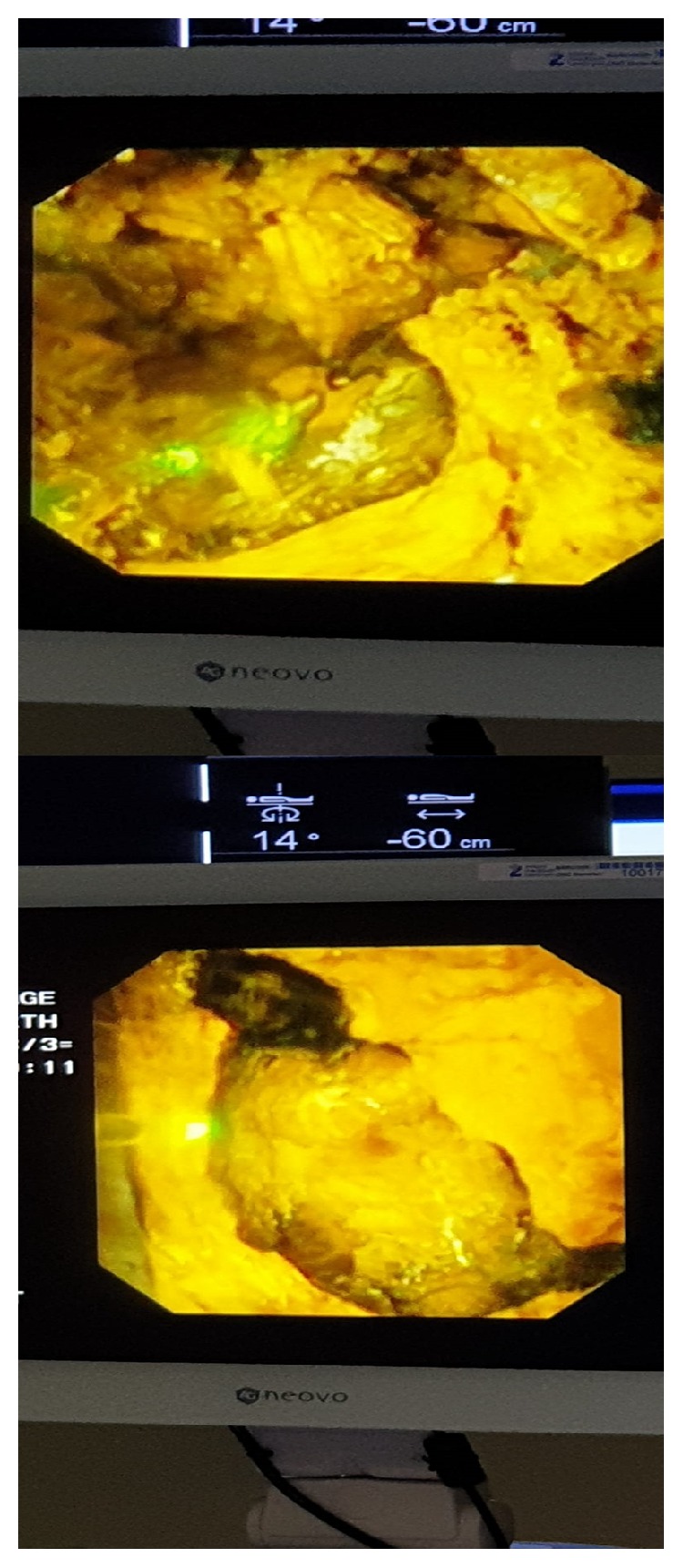
Photographs of the monitor showing the endoscopic view of a large gallstone.

The second patient is also a woman of 77 years. She was known with breast cancer for which she received surgery, radiotherapy, and chemotherapy 14 years earlier. Seven years earlier she underwent ERC because of colics. Ultrasound showed a dilated common bile duct and gallstones in the gallbladder. During ERC a large juxta‐papillary duodenal diverticulum was noticed. Nevertheless canulation of the common bile duct was successful, papillotomy was done, and a stone was removed. Laparoscopic cholecystectomy was done. She did well until the beginning of 2019. This time she was admitted because of fever and jaundice. Laboratory investigations showed signs of cholestasis and inflammation: white blood cells 14.7 × 10*E*9/l; ALAT 163 U/l; total bilirubin 105 *µ*mol/l; conjugated bilirubin 95 *µ*mol/l; GGT 547 U/l; Alkaline Phosphatase 141 U/l; and CRP 120.8 mg/l. Ultrasound showed minimal dilated bile ducts with stones in the common bile duct. ERC was done. This time the procedure was impossible because the diverticulum had increased in size and, despite the previous papillotomy, the papilla could not be canulated anymore. However, due to the manipulations with the canula drainage of bile was restored. The patient was treated with antibiotics because of positive blood cultures with *K. oxytoca* and *S. aureus*. A PTC was done and an internal drain was placed. After improvement of the clinical condition, PTC with assistance of the ureteroscope and laser was performed. During two consecutive procedures large stones (Figure 1) were fragmented and removed through the open papilla. The patient recovered uneventfully.

## 2. Discussion

Percutaneous transhepatic cholangiography can be done in cases of altered anatomy. Especially if patients with stones in the common bile duct had undergone Billroth II resection or Roux‐Y anastomosis it can be very difficult or even impossible to reach the papilla endoscopically with the duodenoscope or even with gastroscope or double balloon endoscope.

The same is true for patients with a gastric bypass.

Various techniques have been reported: a double balloon enteroscope‐assisted ERCP, laparoscopic transgastric ERCP, laparoscopic or open biliary surgery, and interventional radiology [1]. Somasekar et al. did a literature review on the methods used for removing stones from the CBD after bariatric surgery. Overtube‐assisted endoscopy appears to be a popular technique, and 10 studies employing this technique were identified. The success rate for ERCP with this approach is between 60% and 70%. Studies using a combination of radiological and endoscopic techniques report a success rate of 60%–70%, though the endoscopic ultrasound‐guided technique has been reported to have higher success rates (90%–100%). Surgery‐assisted ERCP also appears to be widely reported and has a consistently high ERCP success rate (80%–100%), with an added advantage of the option to perform a concomitant cholecystectomy. There are very few reports on using surgery as the sole option in this scenario [2]. Trans‐gastric approach has been proposed but entails high ERCP‐related risks. The angle in which the endoscopy has to be introduced in the duodenum makes the procedure difficult. Laparoscopy assisted trans‐gastric rendezvous is a one‐step procedure that may lower the risks of these patients [3]. Stage et al. already described a simple percutaneous transhepatic cholelithotripsy procedure [4].

In the present cases an elegant method is used. The equipment is described in Table 1. The urologist can play a role in the treatment of patients with complicated common bile duct stones [5]. Rimon et al. treated 22 patients with large stones successfully with the flexible ureteroscope via percutaneous antegrade access [6]. Maggi et al. also used the holmium laser for removal of biliary stones in liver grafts [7].

The procedure can be done under propofol sedation and local anaesthesia (see technique of the procedure). The percutaneous transhepatic endoscopic approach is a combined procedure with interventional radiology. Antegrade biliary access is achieved, and a wire is placed into the common bile duct. A 12/14 F ureteral access sheath is then placed under fluoroscopic guidance. A flexible ureteroscope is guided through the access sheath into the biliary system [8].

The urologist has great experience with this instrument in the removal of kidney stones. The stones can be fragmented under direct endoscopic vision, and the fragments are flushed or pushed into the duodenum.

It can be concluded that percutaneous endoscopic holmium laser lithotripsy is a minimally invasive alternative to all other techniques published in the literature, especially for open salvage surgery for complex biliary calculi. This treatment is both safe and efficacious. Of course, success depends on a multidisciplinary approach [9, 10].

## Figures and Tables

**Table 1:** Technique of PTC with assistance of the ureteroscope.

*Standard PTC*
Ultrasound guidance
NEFF set: transitional dilator with a hollow stainless steel wire and a Chiba needle with an obturator.
Introduction of a 7 French sheath over a stiff guide wire.
Via the left intra‐hepatic biliary tract catheterisation of the duodenum with vertebral catheter and Terumo angled wire.
For in‐ and external drainage placing of a 10 French biliary drain over stiff wire.

*Flexible ureteroscope*
Olympus URF‐V2
Uropass ureteral access sheat 24 cm, 12/14 French from Olympus, and a continue flow saline 0.9%.
Laserprobe 230 *µ*m Boston Scientific.
Holmium laser StarMedTec: 8 Herz with 800 mJoule.

*Lithotrypsy*
Placing of a second guide wire as safety wire.
Introducing the stiff guide‐wire and placing of a 12/14 French sheath to make introduction of the ureteroscope possible.

*Flushing*
Saline 0.9% 3 L.
